# Immobilized laccase mediated dye decolorization and transformation pathway of azo dye acid red 27

**DOI:** 10.1186/s40201-015-0192-0

**Published:** 2015-04-25

**Authors:** Meenu Chhabra, Saroj Mishra, Trichur Ramaswamy Sreekrishnan

**Affiliations:** Department of Biology, Indian Institute of Technology-Jodhpur, Jodhpur, 342011 Rajasthan India; Department of Biochemical Engineering and Biotechnology, Indian Institute of Technology Delhi, Hauz-Khas, New-Delhi, 110016 India

**Keywords:** Immobilization, Polyvinylalcohol beads, Laccase-mediator, Acid Red 27, Azo dye degradation

## Abstract

**Background:**

Laccases have good potential as bioremediating agents and can be used continuously in the immobilized form like many other enzymes.

**Methods:**

In the present study, laccase from *Cyathus bulleri* was immobilized by entrapment in Poly Vinyl Alcohol (PVA) beads cross-linked with either nitrate or boric acid. Immobilized laccase was used for dye decolorization in both batch and continuous mode employing a packed bed column. The products of degradation of dye Acid Red 27 were identified by LC MS/MS analysis.

**Results:**

The method led to very effective (90%) laccase immobilization and also imparted significant stability to the enzyme (more than 70% after 5 months of storage at 4°C). In batch decolorization, 90-95% decolorization was achieved of the simulated dye effluent for up to 10–20 cycles. Continuous decolorization in a packed bed bioreactor led to nearly 90% decolorization for up to 5 days. The immobilized laccase was also effective in decolorization and degradation of Acid Red 27 in the presence of a mediator. Four products of degradation were identified by LC-MS/MS analysis.

**Conclusions:**

The immobilized laccase in PVA-nitrate was concluded to be an effective agent in treatment of textile dye effluents.

## Introduction

The effluents generated from textile industries are reported to be one of the top ten contaminating sources of water bodies. Ligninolytic enzymes, including laccases, produced by white rot fungi find immense applications in treatment of the toxic wastes generated from textile industries [1,2]. Application of laccases for dye degradation has not been reported so far on an industrial scale mainly because of the high cost of production of these enzymes. In any treatment method, development of a reusable enzyme system, such as that generated by immobilization of enzymes, is most beneficial.

Several attempts have been made to immobilize laccases [3,4]. A number of studies report on the use of immobilized laccase preparations for dye decolorization [4-12]. Immobilization of laccase has been reported to increase thermostability and resistance to inhibitors when compared to the free enzyme [6,7,12]. While most of these studies report the use of immobilized preparations in the batch mode, Palmieri et al. [13] used laccase, immobilized in copper alginate beads, to perform continuous decolorization of dye solutions. However, an additional step that involved coating immobilized enzyme beads with chitosan was added to prevent leaching of laccase during decolorization [13]. Keeping in view the benefits of continuous decolorization and to circumvent additional steps required to reduce laccase leaching, the aim of this study was to immobilize *Cyathus bulleri* laccase. Laccase from this fungus has been demonstrated to decolorize a number of reactive dyes [14,15]. The rates of dye decolorization were significantly enhanced in the presence of mediators [16,17]. This laccase was immobilized in Poly Vinyl Alcohol (PVA) based polymers crosslinked either by nitrate or boric acid. PVA is a reasonably cheap material and its polymers are reported to have high mechanical strength [18]. The PVA immobilized laccase preparation was tested for its applicability in batch and continuous decolorization processes. Immobilized laccase was also used to investigate degradation of an azo dye, namely, Acid Red (AR) 27. AR 27, also known as Amaranth, is an anionic azo dye and is applied to natural and synthetic fibers, paper, leather, and phenol-formaldehyde resins. It was more commonly used as food and cosmetic colorant. However, its use has been banned in USA by food and drug administration (FDA) as it is a suspected carcinogen. The dye is known to be decolorized by treatment with laccase/laccase-mediators [10] but no work has been done to elucidate its breakdown by laccase.

The present study was thus conducted with the following major objectives: (i) to immobilize laccase in PVA based beads and to test its stability and efficacy for both batch and continuous decolorization and (ii) to determine degradation capability of the immobilized laccase + mediator on AR 27 and to characterize the degradation products

## Materials and methods

### Laccase production

Laccase was produced in the culture supernatant of *C. bulleri* by cultivating it in basal liquid medium as described previously [14]. The culture filtrate was concentrated by ultrafiltration using 10 kDa membrane in a stirred cell (Amicon). The specific activity of laccase was 31 U/mg protein using guaiacol as a substrate.

### Laccase immobilization

Laccase was entrapped in beads by two different methods. The materials and gel matrices (sodium alginate, calcium chloride, PVA, boric acid and sodium nitrate) were obtained from Himedia labs and Merck chemicals. The concentrated culture filtrate was mixed thoroughly with PVA-alginate solution. The final concentration of PVA and alginate was 8% and 1% respectively. This solution was centrifuged at 4000 rpm for 5 min to remove air bubbles and filled in a 10 ml syringe. Beads were prepared by extruding the mixture as drops into a stirred saturated boric acid solution to form beads and these were allowed to stay in the same solution for 20 min. The beads were then transferred to 1 M sodium dihydrogen phosphate solution for hardening and further incubated for 1 h. The hardened beads were then extensively washed with distilled water and stored in a minimum amount of water at 4°C. PVA-nitrate beads were prepared by extruding the PVA-alginate-enzyme mixture as drops into stirred 50% (w/v) solution of sodium nitrate containing 1% calcium chloride (w/v). The beads were allowed to stand in this solution for 6 h after which washings with distilled water were performed. The resultant beads were stored in minimum amount of water at 4°C.

The washings were collected to determine laccase activity. Percent immobilization was calculated by subtracting the activity lost in washings from the number of initially loaded units used for immobilization. In order to determine the specific activity, i.e. number of active laccase units/g of beads, the laccase assay was performed at 55°C which resulted in bead dissolution. Total protein was estimated by Lowry’s method.

### Determination of laccase leaching and bead stability

Leaching of laccase from beads was determined by incubating specific number of beads with a fixed initial activity in 5 ml of 50 mM sodium acetate buffer, pH 5.0. The beads were shaken at 100 rpm at 30°C. Enzyme activity was measured in the samples withdrawn after specific time intervals. The final activity was subtracted from the initial activity to determine enzyme loss due to leaching.

Bead stability was determined by incubating 10 intact beads in 2 ml of distilled water in a 25 ml conical flask and stirring at different speeds (ranging from 50–1000 rpm) for 30 min (modified protocol Yujian et al. [19]). The % intact granules v/s rpm plot was obtained for two different kinds of beads. The amount of laccase activity retained by immobilized preparations was determined.

### Characterization of immobilized laccase

Laccase stability, optimum pH, temperature, resistance to some laccase inhibitors (chloride ions, sodium azide and EDTA after 1 h pre-incubation) and kinetic parameters (K_m_ and K_cat_) were determined and compared to that of the free enzyme. Laccase assays were performed using either guaiacol or 2,2’-azino-bis (3-ethylbenzothiazoline-6-sulphonic acid) or ABTS as a substrate as described earlier [14] by adding fixed amount of either free or immobilized laccase. The concentration range of laccase inhibitors used for IC50 calculation ranged from 0–2000 mM NaCl, 0–1000 mM EDTA and 0–50 μM sodium azide. The ABTS concentration range for calculating K_m_ and K_cat_ values was 0–1 mM. The values of catalytic constants were measured using the Lineweaver Burk plot. The value of K_cat_ was determined by dividing V_max_ with total enzyme cocentration [E_tot_].

#### Decolorization experiment using immobilized enzyme

Decolorization experiments were performed using the conditions optimized previously [17]. Decolorization reaction was performed in a 25 ml conical flask. The reaction mixture consisted of either 100 μM of Acid Violet 17 or Basic Green 4 solution (that simulated absorbance of the textile effluent), 100 μM of ABTS and immobilized enzyme (100 mU/ml). The incubation was done at 30°C at 100 rpm. The change in absorbance at 542 nm (λ_max_ of Acid Violet 17) and 618 nm (λ_max_ of Basic Green 4) was used to compute % decolorization. After one cycle of decolorization (~90% decolorization), liquid was withdrawn and fresh dye solution was added to initiate a new cycle of decolorization.

### Continuous decolorization in a packed bed column

**Figure 1:**
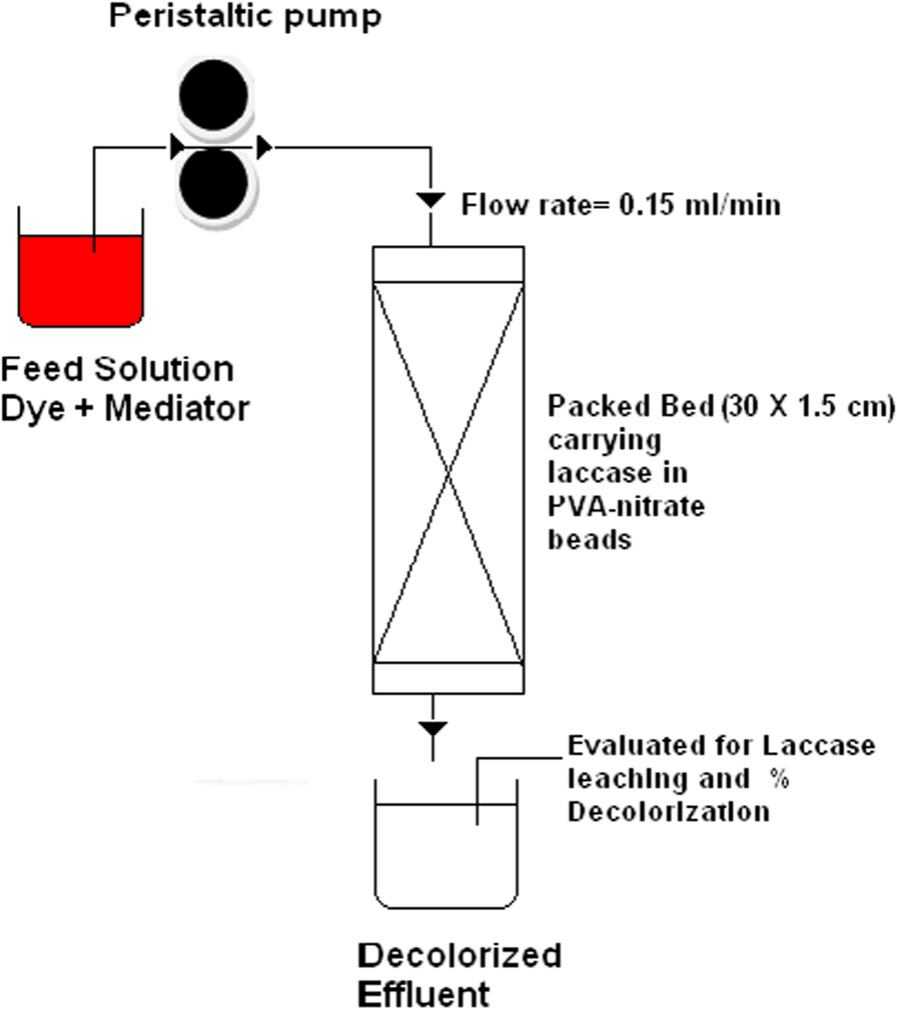
Schematic diagram of the continuous packed column.

Continuous decolorization of the simulated effluent was carried out in a column (30 cm × 1.5 cm) with approximate working volume of 50 ml. The schematic diagram of the same is shown in Figure 1. The simulated effluent contained 100 μM Acid Violet 17, as used in the local textile mills (m/z 710 and also with some unidentified peaks), 2.5 g l^−1^ sodium sulfate as per the specifications for dyeing wool, and 100 μM ABTS in 50 mM sodium acetate buffer (pH 5.5). This feed solution was saturated with oxygen and continuously fed into the column using a peristaltic pump at a flow rate of 0.15 ml/min. This flow rate was selected through initial optimization experiments based on determining an effective hydraulic retention time for decolorization of the dye. The bed was earlier saturated with the dye solution without mediator in order to rule out decolorization due to adsorption on the matrix. Percent decolorization was determined at different time intervals. The samples were also assayed for leached laccase activity.

Degradation of AR 27 (obtained from Sigma Aldrich) was performed using 100 μM AR 27 + 1 mM 1-hydroxybenzotriazole monohydrate (HOBT). The concentration of mediator was selected based on the previous reports [5]. The decolorization reaction was monitored at 515 nm.

### Toxicity and mutagenicity assessment

Toxicity analysis was done by measuring decline in oxygen consumption rate of *Pseudomonas putida.* Mutagenicity was assessed by standard Ames test using *Salmonella typhimurium* TA 98 and 100 strains as described earlier [16].

### Mass analysis

LC-MS analysis was performed for analysis of degradation products of AR 27 using Perkin Elmer HPLC coupled with Qstar Electrospray ionization (ESI) high resolution mass spectrometer (Applied Biosystems, USA) equipped with quadruple and Time of Flight mass analyzers. The decolorized dye samples were introduced into the reverse phase C-18 HPLC column (LiChrospher) using an autosampler at a flow rate of 0.2 ml/min. Elution was performed using a combination of solvent A(acetonitrile) and solvent B (water) in 1:1 ratio. The products were monitored at 210 nm using a UV-Visible detector. The flow coming from HPLC was introduced into the mass spectrometer (MS). The MS was operated in the negative ion mode and other parameters were set as follows: ion spray voltage −4500 V, Nebulizer gas: 35 lb/in^2^, Curtain gas: 25 lb/in^2^, Declustering potential: −80 V, Focussing potential: 265 V. Collision energy was varied from −20 to −80 V for analyzing product ion spectra.

## Results and discussion

### Laccase immobilization, leaching and bead stability

PVA-boric acid and PVA-nitrate gave 65 and 90% immobilization respectively. Leaching of laccase was monitored in both preparations and it was found that PVA-nitrate retained 75% laccase activity after 108 h of incubation at 30°C at 100 rpm (Table 1). Both the bead type were highly stable even up to 1000 rpm. While PVA-boric acid beads exhibited rapid loss in laccase activity, PVA-nitrate beads retained 20% laccase activity even after incubation at 1000 rpm (Table 1).

**Table 1:** **Laccase immobilization and residual laccase activity in immobilized preparations**

**Entrapment method**	**% immobilization**	**% Laccase activity retained after 108 h incubation at 100 rpm, pH 5.5 at 30°C.**	**% Intact beads at 1000 rpm**	**% Laccase activity retained after incubating at 1000 rpm**
PVA-boric acid	65 ± 5	43 ± 2	92 ± 3	0
PVA-nitrate	90 ± 2	73 ± 2	100 ± 0	20 ± 1

The major obstacles in implementing laccase-mediator systems for bioremediation at an industrial scale are the cost of the enzyme and the mediators. Immobilized enzymes allow for an efficient use of available catalyst. Multiple batches of substrates can be processed as the enzyme does not get washed off with the effluent [20]. Also, immobilized enzymes generally exhibit higher resistance to inhibitors and high temperatures. The process of immobilization itself should also be less expensive, easy to perform and effective and this can generally be achieved using cheaper matrices and easy methods of immobilization. Any method which fulfills the above criteria qualifies to be used at large scale making the overall process economical.

The suitability of any immobilized enzyme preparation is largely dependent on the mechanical and chemical stability of the immobilization matrix. In earlier work, immobilization of laccase was carried out in alginate beads cross linked with calcium, copper or zinc. Copper alginate and zinc alginate showed high % immobilization and laccase retention but these preparations were unstable both mechanically and chemically. In the present study, laccase was immobilized in PVA-boric acid and PVA-nitrate beads, making it the first study involving PVA for this enzyme. The immobilization was performed by modifications in the protocol given by Chang and Tseng [21]. Also, this immobilization method fulfilled most of the criteria listed above The beads were found to be stable and of high mechanical strength, a property described earlier also [22]. Also, higher percentage of laccase was immobilized. The beads were stable enough to be used in continuous operations in a column reactor. Palmeiri et al. [13] and Brandi et al. [23] reported 65% immobilization of laccase in copper alginate beads. Lower values for immobilization also mean using higher quantities of initial enzyme making the process expensive [24]. Liu et al., [25] reported 91% retention of laccase activity during immobilization on carbon based mesoporous magnetic composites. The preparation of such composites, however, involved complicated procedures while entrapment by far is the easiest way to immobilize enzymes.

#### Characterization of immobilized laccase

It was found that immobilization of laccase did not confer any increased resistance to common inhibitors such as chlorides, EDTA and azide. The IC50 values were nearly equal to that of the free enzyme. The immobilized laccase, however, displayed lower affinity (K_m_) for ABTS compared to the free enzyme. The K_cat_ values also decreased for the immobilized enzyme (Table 2). This observation has been made with several other immobilized enzyme preparations [4,23] and is attributed to mass transfer resistance displayed by enzymes in the entrapped state. This eventually leads to a decline in catalytic efficiency. In our study, this can also be attributed to the low permeability of PVA beads leading to low diffusivity of dyes as well as oxygen required for laccase action. The storage stability of the immobilized enzyme, however, was substantially increased as compared to the free enzyme and is the highest reported till now. The only other highly stable laccase was reported by immobilization in controlled porosity silica beads [26].

**Table 2:** **Comparison of properties of immobilized and free laccase**

**Parameter**	**PVA-nitrate immobilized laccase**	**Free laccase**
IC 50 for chloride	450 ± 22 mM	600 ± 33 mM
IC 50 for EDTA	250 ± 17 mM	250 ± 10 mM
IC 50 for sodium azide	6 ± 0.3 μM	5 ± 0.5 μM
K_m_ (ABTS)	65 ± 4 μM	37 ± 2.1 μM
K_cat_ (ABTS)	1675 ± 75 sec^−1^	2996 ± 179 sec^−1^

The enzyme stability increased for immobilized preparations with more than 80% activity remaining after 4 months of storage at 4°C. Free laccase, on the other hand, exhibited complete loss in activity in a period of less than a month. The optimum pH and temperature of the laccase remained the same with ABTS for immobilized preparations as that for the free enzyme.

### Batch decolorization using the immobilized laccase

**Figure 2:**
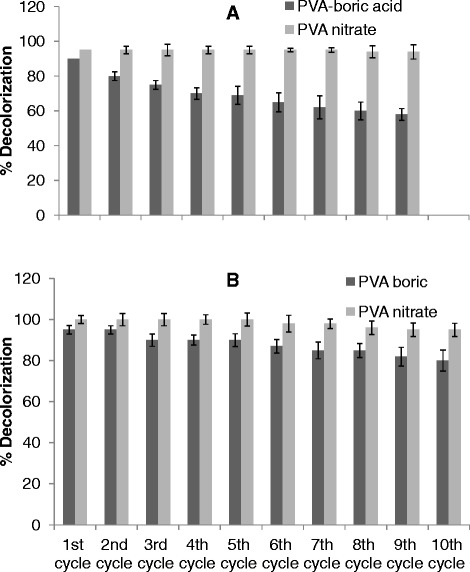
Batch decolorization of simulated effluent using PVA-boric acid and PVA-nitrate entrapped laccase. **(A)** Acid violet 17 or **(B)** Basic Green 4.

In order to determine the decolorization efficiency of the immobilized enzyme in batch mode, an acidic and a basic dye were selected. Acid Violet 17 decolorized at a much slower rate compared to Basic Green 4 [17]. Therefore, both the dyes offered different chemical structure and decolorization kinetics making them appropriate to determine the behavior of immobilized laccase. Decolorization of two different simulated effluents, one containing Acid Violet 17 and the other containing Basic Green 4 was performed. PVA-nitrate beads were chemically stable in both the dye solutions and led to 95% decolorization of Basic Green 4 even up to 20 cycles. In batch mode, the preparations also led to 90% decolorization of the effluent containing Acid Violet 17 up to 10 cycles (Figure 2A and B). The results were promising when compared to previous reports wherein the immobilized laccase was not highly reusable in batch studies. This was largely due to loss in laccase activity due to leaching or inactivation [13,23].

### Continuous decolorization using immobilized laccase

**Figure 3:**
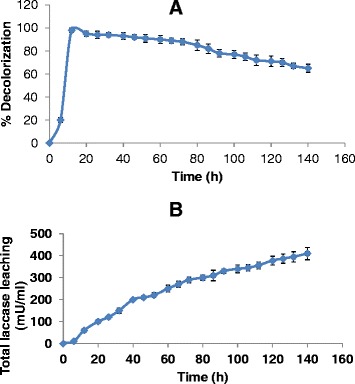
Continuous decolorization in packed bed column using PVA-nitrate beads. **(A)** Profile of dye decolorization with time and **(B)** Leaching of laccase from the column.

The immobilized laccase preparations were used to perform decolorization of the simulated effluent. The decline in % decolorization achieved in packed bed column was directly correlated with remaining laccase activity in the beads. PVA-nitrate packed bed retained 60% of the initial laccase activity even after 120 h of use. The decolorization of the simulated effluent was also more than 70% up to 120 h (Figure 3A and B).

### AR 27 decolorization and degradation using immobilized laccase

**Figure 4:**
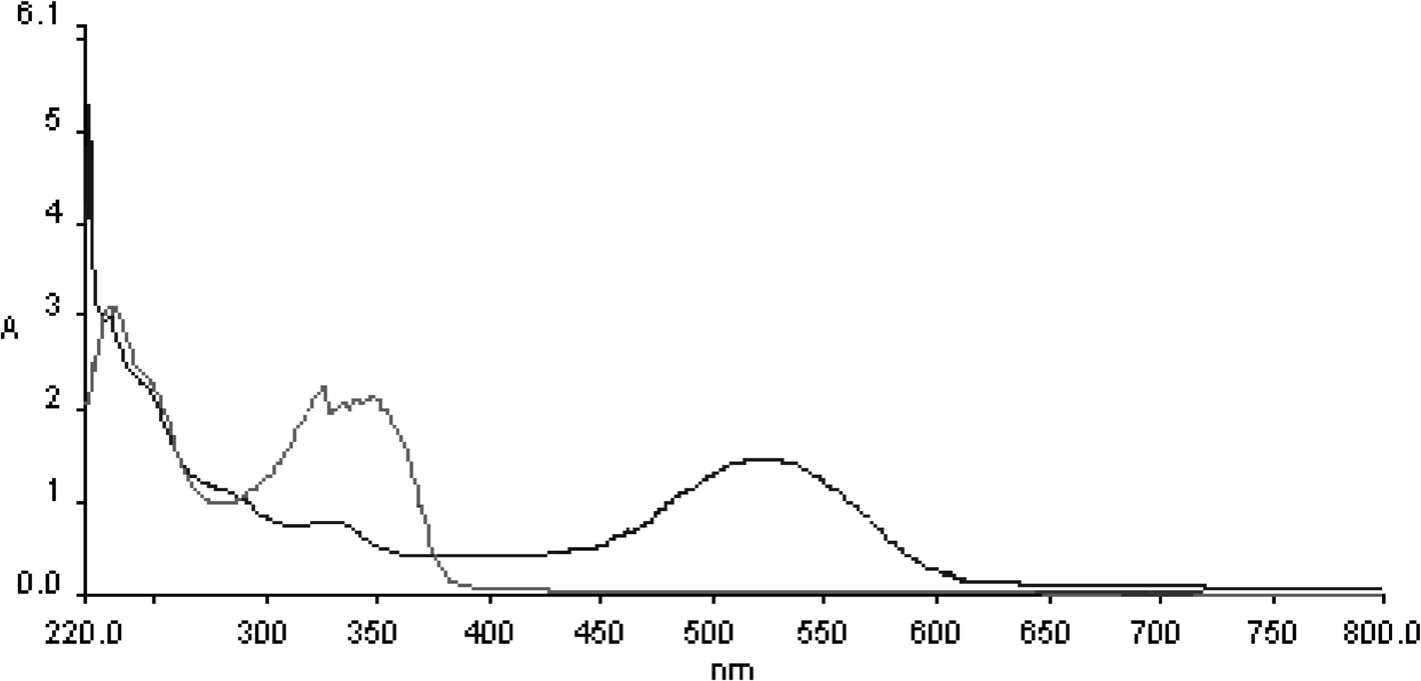
Absorption profile of AR 27 (―) and AR 27 treated with laccase and 1-HOBT (―).

AR 27 was decolorized by laccase only in the presence of the mediator, as is characteristic of most high redox dyes. Mass spectra of the dye gave multiple peaks which were attributed to sodium and potassium adducts of the dye. Differential ionization of the dye molecule gives multiple peaks at different m/z. The analysis of the decolorized samples led to identification of four products, named from I to IV (Table 3). These persistent products were Compound I: 4-((2-oxo-3, 6-disulfo-2,3-dihydronaphthalen-1-yl)diazenyl)naphthalene-1-sulfonate at m/z 535.9, compound II: 4-diazenylnaphthalene-1-sulfonate at m/z 235.01, compound III: naphthalene-1-sulfonate at m/z 207.0, and compound IV: 3,4-dioxo-7-sulfo-2,3,4,4a-tetrahydronaphthalene-2-sulfonate at m/z 316.94. The UV-visible absorbance scan showed complete disappearance of peaks in the visible range (Figure 4).

**Table 3:** **Summary of the degradation products of Acid Red 27 after laccase-mediator action as observed by ESI-MS/MS**

**Product**	**Mass/charge (m/z)**	**Major product ion peaks as observed in MS/MS spectrum**	
		**Collision Energy (V)**	**m/z (% Relative intensities)**
Dye alone	[M-H]- =536.97	−50	316.97 (100); 301.96 (50); 237.02 (40) 206.00 (30); 79.96 (30); 518.96 (20); 457.02 (10); 439.00 (10); 377.07 (5) 365.09 (5).
I	[M-H]- =535.9	−50	315.97 (100); 301.96 (50); 236.02 (40) 206.00 (30); 79.96 (30); 518.96 (20); 457.02 (10); 439.00 (10); 377.07 (5) 365.09 (5)
II	[M-H]- =235.01	−30	182.65 (100); 233.99 (45); 79.95 (30); 219.99 (20).
III	[M-H]- =207.01	−30	207.01 (100); 183.01 (50); 79.95 (40); 119.05 (20)
IV	[M-H]- =316.94	−40	316.9 (100); 289 (50); 245.06 (20); 79.95 (15); 255.20 (10); 145.0310 (10); 236.98 (5).

**Figure 5:**
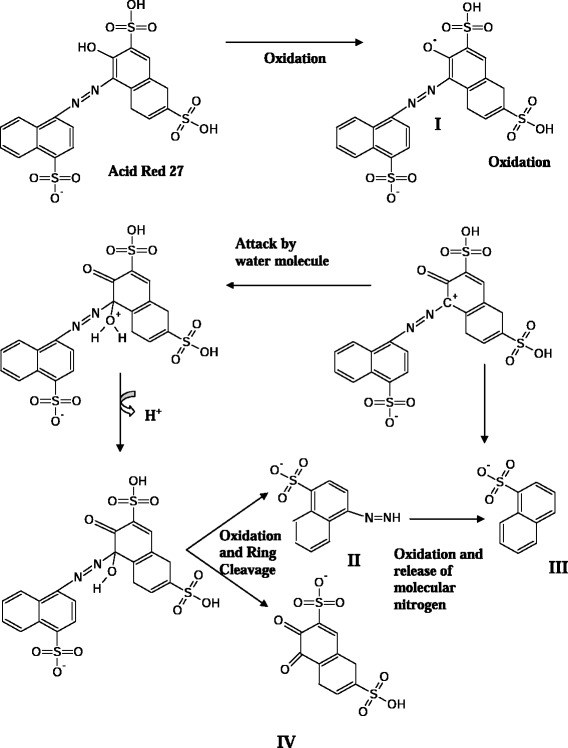
Proposed pathway of decolorization of AR 27 using laccase-mediator system.

Figure 5 shows the proposed decolorization pathway of AR 27 by laccase-mediator. The four degradation products which were identified are proposed to appear by the action of laccase and mediator in the following way. Compound I at m/z 535.9 resulted from the oxidation of the phenolic dye group to a quinine. This laccase-mediator assisted two electron oxidation was proposed to occur via phenoxy radical formation and is one of the important intermediates through which the dye undergoes further conversion. This is followed by a nucleophilic attack by a water molecule which creates a site for further action of laccase/mediator system. This explains the formation of other identified products. Compound I undergoes further oxidation resulting in splitting of the dye molecule and generation of compound II at m/z 235.01 and compound IV at m/z 316.94. Compound II gets further oxidized to compound III which was detected at m/z 207.0. The nitrogen in the azo bond was thus released as molecular nitrogen. This form of azo bond disruption was earlier proposed by Chivukula and Renganathan [27]. The peak representing [SO_3_] ^–^ at m/z 79.95 did appear in the decolorized dye spectrum. However, this was not attributed to laccase action as it also appeared in the spectrum taken with dye alone, although at slightly lower intensities. Thus, the appearance of these peaks is possibly due to ionization induced fragmentation of the dye molecule or its degradation products.

The high molecular weight polymerization products were absent which otherwise appear on treatment of dyes with free laccase on slightly long incubations. Zille et al. [28] observed high molecular weight molecules which were the polymerization products of intact dye and its degradation products obtained on longer incubations with free laccase.

### Toxicity and mutagenicity assessment

**Figure 6:**
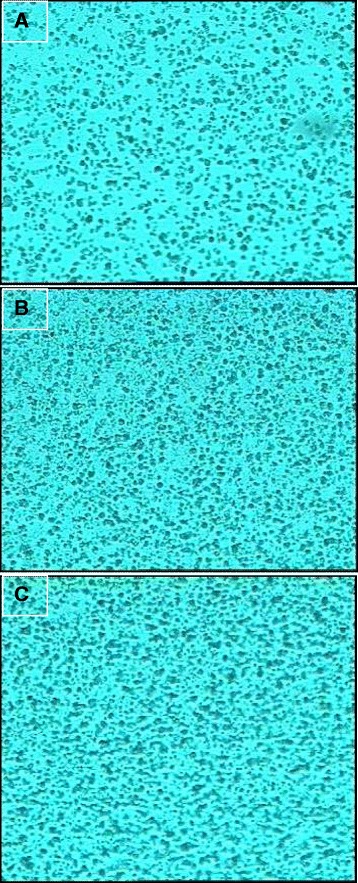
Background lawn of *S. typhimurium* TA 98 obtained during Ames test. **(A)** Untreated AR 27, **(B)** AR 27 treated with laccase and 1-HOBT, **(C)** Negative control-Distilled water.

AR 27 did not inhibit respiratory activity in *P. putida* but was found to be mutagenic as assessed by the Ames test. Similar observations have been made earlier [29]. Also, slight thinning of the background lawn of *S. typhimurium* occurred showing cytotoxic effect (Figure 6). Treatment with laccase-mediator did not add any toxicity but led to reduction in mutagenicity and also restored density of background lawns in the Ames test. This is possible as the mutagenic azo bond is disrupted by laccase-mediator action.

## Conclusions

Laccase immobilized in PVA-nitrate was found to be stable for use in several decolorization studies using batch and immobilized systems. High mechanical strength of the beads allowed continuous operations in a column reactor. Immobilized laccase was also used for decolorization and degradation of the azo dye AR 27. The dye degradation studies showed that AR 27 underwent degradation to smaller moieties and a pathway of degradation was proposed. It was also concluded that polymerization products appearing during dye decolorization can be avoided through continuous decolorization process by providing just appropriate hydraulic retention time.
